# Efficacy of Phenobarbital and Prognosis Predictors in Women With Epilepsy From Rural Northeast China: A 10-Year Follow-Up Study

**DOI:** 10.3389/fneur.2022.838098

**Published:** 2022-02-16

**Authors:** Chaojia Chu, Nan Li, Rui Zhong, Danyang Zhao, Weihong Lin

**Affiliations:** Department of Neurology, Neuroscience Center, The First Hospital of Jilin University, Changchun, China

**Keywords:** adult female epilepsy, phenobarbital, curative effect, prognostic predictors, rural Northeast China

## Abstract

**Objective:**

To investigate the efficacy of phenobarbital (PB), factors associated with it, reasons for early treatment termination, and mortality rates in adult women living in rural Northeast China.

**Methods:**

A prospective study was conducted in seven counties of Jilin Province from 2010 to 2020. Adult women diagnosed with convulsive epilepsy were recruited into the study and baseline demographics recorded upon enrollment. Seizure frequency, prescribed drug dose, and adverse reactions were monitored monthly by door-to-door survey or telephone interview.

**Results:**

A total of 1,333 women were included in the study. During the follow-up period, 169 participants (12.7%) were lost to follow-up, and 100 of them (7.5%) died. The percentage of seizure-free participants was 45.3% in the first year, 74.6% in the third year, and 96.6% in the 10th year. A higher baseline seizure frequency (OR = 1.005, 95% CI: 1.002–1.009), more frequent loss-of-consciousness seizures (OR = 1.620, 95% CI: 1.318–1.990), a higher daily dose of PB in the first year (OR = 1.018, 95% CI: 1.014–1.022), a younger age at onset (OR = 0.990, 95% CI: 0.982–0.998), and more severe drowsiness (OR = 1.727, 95% CI: 1.374–2.173) were associated with an increased risk of seizures in the first year, and the higher baseline seizure frequency was still associated with the occurrence of seizures in the third (OR = 1.007, 95% CI: 1.004–1.010) and fifth year (OR = 1.005, 95% CI: 1.002–1.008). Age at enrollment (HR = 0.983, 95% CI: 0.971–0.994) was the only factor that correlated with withdrawal from the study and with the death of the participant during the follow up period, but the correlation in each case was in opposite directions.

**Significance:**

PB has high effectiveness, retention rate, mild side effects, and tolerability when used as a treatment for epilepsy in women from rural areas. Baseline seizure frequency is an important predictor of prognosis regardless of treatment duration. PB is still a valuable tool for the management of epilepsy in adult women from poverty-stricken areas.

## Introduction

Epilepsy is a common neurological disorder that affects ~65 million people worldwide (1). It affects people of every age and background, and its incidence tends to be lower in women, with an estimated prevalence of 6.85 cases per 1,000 women (2). Differences in the structure and network connections between the male and the female brain may contribute to this differential susceptibility (3). Some studies have also suggested that women may be more likely to conceal their epilepsy diagnosis if they live in regions where this could jeopardize their marriage prospects or cause social marginalization (4). Epilepsy in women with are complex due to their association with periodic changes in hormone levels, cytochrome P450 activity, neurotransmitter systems, and biological differences in neuronal networks (5, 6). Moreover, some specific problems that women face during their lifetime, such as pregnancies and fertility issues, make treatment decisions for them more personalized and difficult. In addition to the impact of the disease itself, adults with epilepsy may also suffer from psychological and emotional consequences, including anxiety, depression, stigmatization, and social embarrassment (7). They may also experience difficulties at work, in their marital and family life, and moving around independently (8). Medical expenditure and productivity losses directly and indirectly increase the burden on the family and on society (9). Seizure remission manifestly ameliorates most social implications of epilepsy for social life, with the exception of leisure activities and marital status (8).

The burden of epilepsy is not evenly distributed. The prevalence of epilepsy in low- and middle-income countries is roughly twice that in high-income countries (2, 10). Moreover, there is a huge gap in the treatment of epilepsy between different regions of the world (11), The treatment gap is over 75% in low-income countries and over 50% in most lower middle- and upper middle-income countries, while many high-income countries have gaps >10%. The treatment gap varies widely, not only between countries but also within them, being significantly higher in rural areas (12), It was estimated that 63% of people with active convulsive epilepsy (ACE) in the northern and eastern areas of China never received appropriate treatment (13). The treatment gap in women with ACE was estimated to be 66.0% and tended to be higher in those over 20 years of age in rural Western China (14). These numbers suggest that a large number of female patients in rural, low-income areas do not receive an effective and regular treatment, and it is, therefore, crucial to provide them with inexpensive, efficacious, accessible, and regular therapy.

As a member of a project to reduce the treatment gap launched by WHO, the International League Against Epilepsy (ILAE), and the International Bureau for Epilepsy, China began the free distribution of phenobarbital (PB) in rural areas in 2000 (15). In recent years, many studies have focused on the special characteristics of epilepsy in women and the quality of life of patients. However, improving control of the disease in low-income areas is still of primary importance and cannot be neglected. This prospective cohort study collected 10-year follow-up data to investigate the efficacy of PB treatment in adult women in rural Northeast China, risk factors associated with efficacy in different periods of treatment, and adverse reactions to the drug.

## Materials and Methods

### Participants

**Figure 1:**
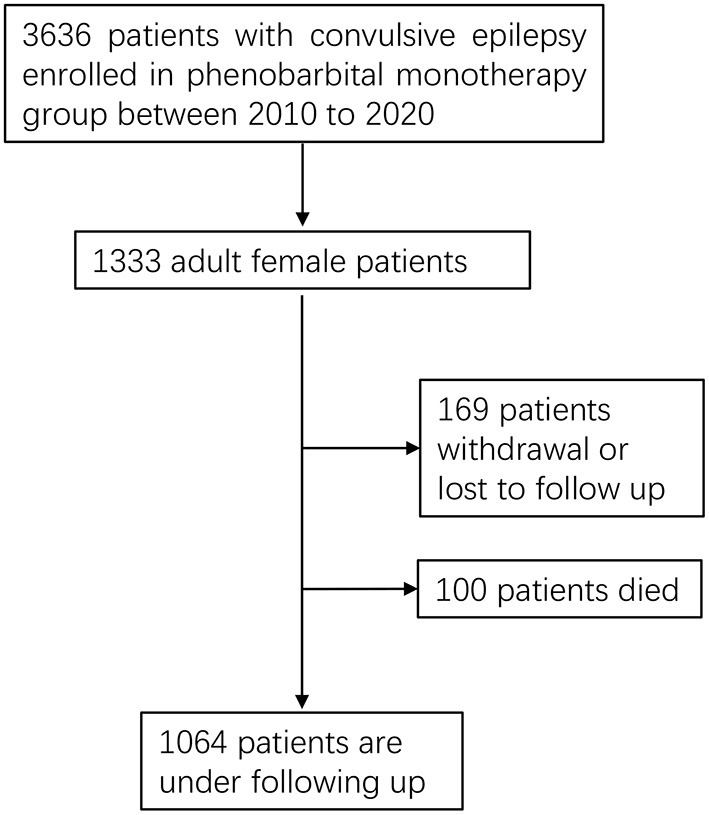
Flow diagram of participant data.

We conducted a prospective study in seven counties of the Jilin Province in Northeast China from 2010 to 2020. Adult women (above 18 years of age) diagnosed with convulsive epilepsy were recruited in our study (Figure 1). The diagnosis of convulsive epilepsy was based on the manifestation of two of the following symptoms: loss of consciousness, rigidity, or generalized convulsive movements; and at least one of the following symptoms: urinary incontinence, bitten tongue or an injury sustained in a fall, post-seizure fatigue, or headache or muscle aches after a seizure (15). In addition to being female and above 18 years of age, patients meeting the following criteria were enrolled into the study and treated with PB: presentation of at least two unprovoked (reflex) seizures occurring more than 24 h apart up to 1 year before enrollment; convulsive seizures in addition to other types of seizures; and no previous treatment or irregular treatment with PB. The exclusion criteria were as follows: (1) seizures associated only with pregnancy, alcohol or drug reduction, (2) allergy to PB, (3) progressive neurological disease diagnosis, (4) history of epileptic status, or (5) current effective treatment with an antiepileptic drug. All participants received PB monotherapy. Patients also need to be reviewed by a neurologist to confirm the diagnosis of epilepsy after initial enrollment. The study was approved by the Ethics Committee of the First Hospital of Jilin University, and all patients provided written informed consent for participation.

### Follow-Up and Data Collection

The study participants were monitored once a month by door-to-door survey or by telephone interview. The monitors recorded demographics at baseline, seizure frequency, prescribed drug dose, and adverse reactions, including drowsiness, ataxia, dizziness, headache, hyperactivity, skin rash, gastrointestinal complaints, and anxiety/depression. Each adverse reaction episode was categorized as mild (unconfirmed and tolerable), moderate (confirmed and tolerable), or serious (confirmed and intolerable). In the event of the death of a participant, the recorded cause was based on the death certificate and a verbal autopsy (16) conducted by expert clinicians through an interview with the family or neighbors of the deceased. The cause of death was classified according to the 10th Edition of the International Classification of Diseases.

### Statistical Analysis

The Shapiro-Wilk test was used to evaluate continuous variables for normality. Values for continuous variables were expressed as median (25th, 75th percentiles) and values for categorical variables as frequencies (%). Wilcoxon Mann-Whitney test was used to compare continuous variables of skewed distribution and rank variables, such as baseline age, onset age, baseline seizures frequency, the dosage of PB, adverse events, and so on. We defined the seizure-free status as the absence of seizures for more than 12 consecutive months and baseline seizures frequency as the number of seizures in the year prior to enrollment. A logistic regression model analysis was applied to identify risk factors for seizure-free status at different stages of follow-up. Cox regression model analysis was applied to identify risk factors for study withdrawal and death. All *p*-values were estimated in a two-tailed manner. Differences with a *p* < 0.05 were considered statistically significant. The data were analyzed using the SPSS software package for Windows, Version 25.0 (SPSS Inc., Chicago, IL, USA).

## Results

### Demographics

We enrolled 1,333 consecutive adult female patients who met the inclusion criteria. PB monotherapy was provided after enrollment. Demographic characteristics and baseline data are shown in Table 1.

**Table 1:** Demographic characteristics of the patients with convulsive epilepsy, *N* (%) or median (minimum–maximum).

	**Total**	**Withdrawal**	**Death**
Number	1,333	169 (12.7)	100 (7.5)
Baseline age, years	43 (18–89)	42 (18–76)	54.50 (18–86)
Onset age, years	20 (0–86)	18 (1–65)	23.50 (0–86)
Baseline seizures frequency per year	10 (2–900)	6 (2–200)	11.50 (2–360)
Duration, months	20 (0–64)	18 (0–50)	20 (0–55)
Follow-up time, months	71 (1–120)	13 (1–106)	29.50 (1–114)
Baseline BMI	22.9 (14.3–57.5)	22.9 (16.3–34.4)	23.2 (14.3–38.3)

### Efficacy and Adverse Reactions of PB Treatment

The effectiveness of PB treatment was graded according to three categories in terms of seizure frequency: seizure-free status for a year, seizure reduction equal to or above 50% from baseline (both considered effective) and seizure increase or reduction below 50% (considered ineffective).

Table 2 shows the changes in seizure frequency derived from PB monotherapy in the first year (*n* = 1,210), third year (*n* = 968), fifth year (*n* = 845), seventh year (*n* = 358), and tenth year (*n* = 59). In the first year of follow-up, the therapy was effective for nearly three quarters of the patients (893 patients, 73.8%). Nearly half of the participants (45.3%) were seizure-free in the first year, and 722 (74.6%) of them were seizure-free in the third year. For longer follow-up times, the proportion of seizure-free patients gradually increased. Of the 59 patients that completed the 10-year follow-up period, 57 of them (96.6%) were seizure-free in the 10th year.

**Table 2:** The changes in convulsive seizure frequency compared to baseline and the daily dosage of PB, *N* (%) or median (P25, P75).

	**The first year (***n*** = 1,210)**	**The third year (***n*** = 968)**	**The fifth year (***n*** = 845)**	**The seventh year (***n*** = 358)**	**The tenth year (***n*** = 59)**
Seizure-free	548 (45.3%)	722 (74.6%)	703 (83.2%)	320 (89.4%)	57 (96.6%)
≥50% reduction in seizure frequency (excluded seizure-free)	345 (28.5%)	144 (14.9%)	86 (10.2%)	21 (5.9%)	1 (1.7%)
<50% reduction in seizure frequency	317 (26.2%)	102 (10.5%)	56 (6.6%)	17 (4.7%)	1 (1.7%)
Daily dosage of PB (mg/day)	90 (60, 120)	90 (60, 120)	90 (60, 120)	90 (60, 127.5)	90 (60, 120)

**Figure 2:**
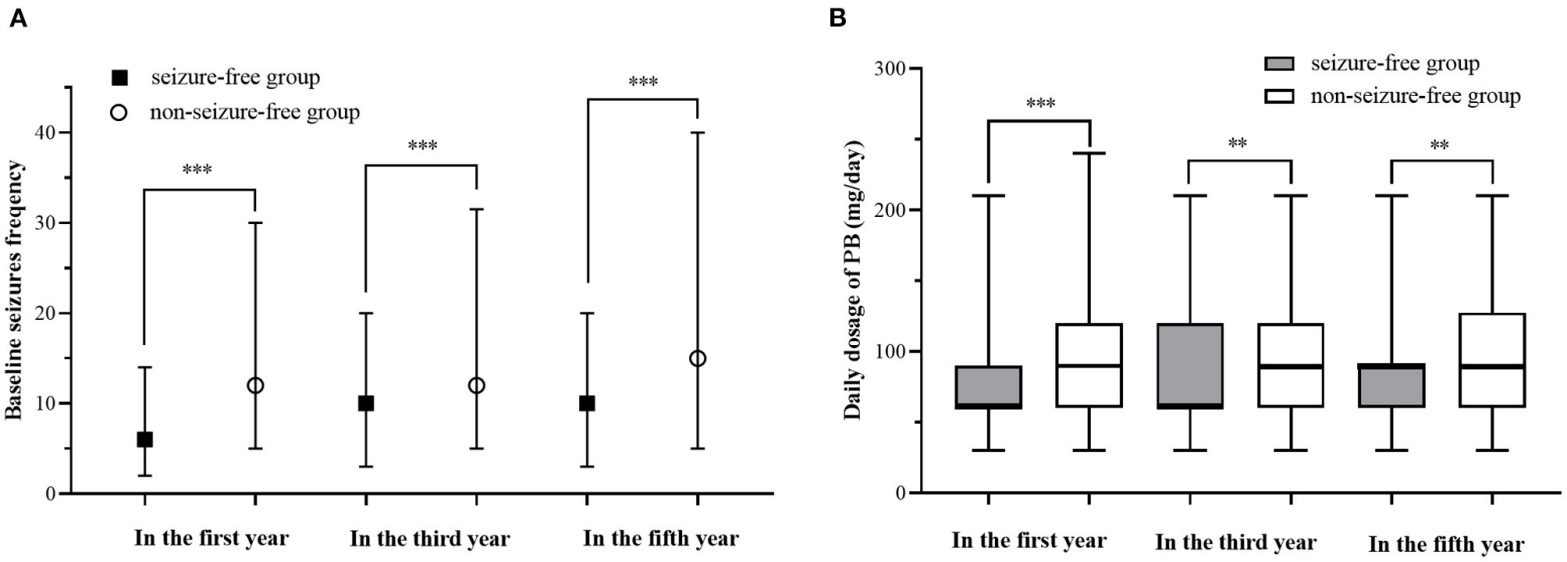
Comparison of baseline Seizures frequency and daily dosage in the first year between different prognosis. **(A)** Comparison of baseline Seizures frequency of different prognosis. **(B)** Comparison of baseline Seizures frequency of different prognosis. “**” indicates a statistical significance (*P* < 0.01) between seizure-free group and non-seizure-free group, “***” indicates a statistical significance (*P* < 0.001) between seizure-free group and non-seizure-free group.

We compared the seizure-free group with the group that had experienced seizures in the first year, third year, and fifth year (Table 3, Figure 2). There were significant differences (*p* < 0.05) between the two groups in the first year of follow-up for several variables: baseline seizure frequency, age of onset, duration before treatment, degree of consciousness, daily dose of PB in the first year, and degree of adverse events. In the third and fifth year, baseline seizure frequency and daily dose of PB in the first year were also significantly different between the two groups. When the variables with significant differences were used as independent variables in a regression analysis (Table 4), we found that higher baseline seizure frequency (OR = 1.005, 95% CI: 1.002–1.009), more frequent unconsciousness (OR = 1.620, 95% CI: 1.318–1.990), a higher daily dose of PB in the first year (OR = 1.018, 95% CI: 1.014–1.022), younger onset age (OR = 0.990, 95% CI: 0.982–0.998), and more severe drowsiness (OR =1.727, 95% CI: 1.374–2.173) were associated with an increased risk of seizures in the first year. Higher baseline seizure frequency still had an effect on the occurrence of seizures in the third (OR = 1.007, 95% CI: 1.004–1.010), and fifth years (OR = 1.005, 95% CI: 1.002–1.008). Other factors that significantly influenced this outcome in the third and fifth years are shown in Table 4.

**Table 3:** Comparison of baseline characteristics, medication and adverse events between different prognosis, *N* (%) or median (minimum–maximum).

	**In the first year**				**In the third year**				**In the fifth year**		
	**Seizure-free** **(***n*** = 548)**	**Non-seizure-free** **(***n*** = 662)**	***z***	***P*** **-value**	**Seizure-free** **(***n*** = 722)**	**Non-seizure-free** **(***n*** = 246)**	***z***	***P*** **-value**	**Seizure-free** **(***n*** = 703)**	**Non-seizure-free** **(***n*** = 142)**	***z***	***P*** **-value**
Baseline age, years	45 (18–86)	42 (18–84)	−1.594	0.111	43 (18–86)	42 (18–84)	−0.814	0.416	42 (18–77)	42 (18–71)	–0.731	0.465
Onset age, years	20 (0–86)	18 (0–73)	−4.291	<0.001	19 (0–80)	18 (0–71)	−0.733	0.464	19 (0–71)	19.5 (0–71)	–0.046	0.963
Baseline Seizures frequency per year	6 (2–400)	12 (2–900)	−8.331	<0.001	10 (2–360)	12 (2–900)	−5.178	<0.001	10 (2–500)	15 (2–900)	−4.256	<0.001
Duration before treatment, months	19 (0–56)	22 (0–64)	−3.894	<0.001	20 (0–56)	21 (0–55)	−0.643	0.520	21 (0–54)	19 (0–52)	–0.511	0.609
Baseline BMI, kg/m^2^	23.0 (15.6–57.5)	22.9 (14.3–49.3)	−0.021	0.983	23.2 (14.3–49.3)	22.7 (15.8–57.5)	−1.471	0.141	23.1 (15.6–44.4)	23.3 (14.3–57.5)	–0.516	0.606
Daily dosage in the first year (mg/day)	60 (60, 90)	90 (60, 120)	−13.606	<0.001	60 (60, 120)	90 (60, 120)	−3.345	0.001	90 (60, 90)	90 (60, 127.5)	−3.326	0.001
Unconsciousness at seizure			−5.640	<0.001			−4.234	<0.001			–0.212	0.832
None	32 (5.8)	41 (6.2)			48 (6.6)	13 (5.3)			41 (5.8)	9 (6.3)		
Always	216 (39.4)	146 (22.1)			232 (32.1)	44 (17.9)			205 (29.2)	39 (27.5)		
Every time	300 (54.7)	475 (71.8)			442 (61.2)	189 (76.8)			457 (65.0)	94 (66.2)		
Adverse eventsy*												
Drowsiness			−3.242	0.001			−0.070	0.944			−2.153	0.031
Ataxia			–.570	0.569			−0.079	0.937			−2.494	0.013
Dizziness			−2.260	0.024			−2.002	0.045			−2.427	0.015
Headache			−1.780	0.075			−0.756	0.450			−1.944	0.052
Hyperactivity			−0.147	0.883			−0.886	0.376			−2.249	0.025
Skin rash			−0.104	0.918			−1.089	0.276			−1.902	0.057
Gastrointestinal complaints			−0.277	0.782			−0.369	0.712			−1.079	0.281
Anxiety or depression			−1.999	0.046			−1.318	0.187			−1.516	0.130

* *Each adverse reaction is divided into none, mild, moderate, or serious. Proportion of each level could be seen in Supplementary Materials for details*.

**Table 4:** Multivariate logistic regression analysis to explore the predictive factors of non-seizure-free at different stages of follow-up.

	**In the first year**			**In the third year**			**In the fifth year**		
	**B**	**OR (95% CI)**	***P*** **-value**	**B**	**OR (95% CI)**	***P*** **-value**	**B**	**OR (95% CI)**	***P*** **-value**
Baseline seizures frequency	0.005	1.005 (1.002–1.009)	0.005	0.007	1.007 (1.004–1.010)	<0.001	0.005	1.005 (1.002–1.008)	0.001
Unconsciousness at seizure	0.482	1.620 (1.318–1.990)	<0.001	0.499	1.648 (1.252–2.169)	<0.001			
Daily dosage in 1st year	0.018	1.018 (1.014–1.022)	<0.001				0.006	1.006 (1.002–1.010)	0.001
Onset age	−0.010	0.990 (0.982–0.998)	0.013						
Drowsiness	0.547	1.727 (1.374–2.173)	<0.001						
Dizziness				0.334	1.397 (1.072–1.820)	0.013			
Ataxia							−0.398	0.672 (0.460–0.982)	0.040

As shown in Table 5, drowsiness was the most common adverse event (58.6%), but most cases were mild (55.2%). This was followed by dizziness (42.1%) and ataxia (26.3%). Skin rash (10.3%) was the least common adverse event.

**Table 5:** Adverse events, *N* (%).

	**None**	**Mild**	**Moderate**	**Serious**
Drowsiness	552 (41.4%)	736 (55.2%)	32 (2.4%)	13 (1.0%)
Ataxia	969 (72.7%)	339 (25.4%)	20 (1.5%)	5 (0.4%)
Dizziness	772 (57.9%)	531 (39.8%)	25 (1.9%)	5 (0.4%)
Headache	905 (67.9%)	405 (30.4%)	18 (1.4%)	5 (0.4%)
Hyperactivity	1,110 (83.3%)	209 (15.7%)	8 (0.6%)	6 (0.5%)
Skin rash	1,196 (89.7%)	130 (9.8%)	3 (0.2%)	4 (0.3%)
Gastrointestinal complaints	1,130 (84.8%)	191 (14.3%)	3 (0.2%)	9 (0.7%)
Anxiety or depression	1,087 (81.5%)	234 (17.6%)	6 (0.5%)	6 (0.5%)

### Withdrawal

During the follow-up period, 169 patients (12.7%) were lost to follow-up for various reasons, among which non-adherence to the prescribed PB treatment was the most common (51.5%). This was followed by relocation away from the area covered in the study (29%) withdrawal due to the development of adverse events (15.4%), pregnancy planning (3%), and treatment termination caused by other diseases (1.2%). A Cox regression was used to analyze the risk factor of withdrawal from therapy, with baseline age at enrolment, age at onset, seizure frequency, degree of consciousness, and degree of adverse events set as independent variables. Age at enrolment (HR = 0.983, 95% CI: 0.971–0.994, *P* = 0.004) was the only variable that significantly influenced the risk of withdrawal.

### Death

A total of 100 patients died during the entire follow-up period (Table 6). Heart disease (29%) and stroke (27%) were the most common causes of death, followed by CNS and non-CNS cancers (10%), unknown causes (9%), accidental death (8%), suicide (7%), other system diseases (7%), and SE deaths (3%). When age at enrolment, age at onset, baseline seizure frequency, duration before enrolment, and degree of consciousness were used as independent variables for a multivariate Cox regression analysis, age at enrolment (HR = 1.057, 95% CI: 1.041–1.073, *P* < 0.001) was found to be the only significant risk factor for death.

**Table 6:** Causes of death in adult female people with convulsive epilepsy in rural Northeast China.

	***N***	**%**
Total	100	100
Cardiac disease	29	29
Stroke	27	27
Accident	8	8
Cancer	10	10
Other tumors (CNS excluded)	7	7
Intracranial neoplasm	3	3
Suicide	7	7
Other system diseases	7	7
SE	3	3
Unknown	9	9

*CNS, Central nervous system; SE, Status epilepticus*.

## Discussion

Monotherapy with standard antiepileptic drugs is still recommended by WHO as a treatment for convulsive epilepsy in children and adults. PB is still the most cost-effective drug for the treatment of epilepsy and can help reduce the treatment gap in economically underdeveloped areas. Many studies indicate that PB will still have a prominent role to play in the future (17, 18).

Epilepsy has different characteristics in different genders, which may be associated with the difference in brain development, hormone levels, P450 activity between females and males (6). Some studies suggest that specific subtypes of epilepsy may have a higher incidence in certain genders (19, 20). Patients' responses to drugs are also different due to gender differences. An animal experiment showed that barbiturates may have higher blood concentrations in females (21). For women with fertility potential, some antiepileptic drugs pose a risk of teratogenesis in their offspring. However, for women without need to conceive, especially in low-income areas, cheap and effective anti-epileptic drugs are still in great demand. In some low-income areas in China, the treatment gap for women is higher than that for men (14). Therefore, we need to pay more attention to female patients. In addition to their special characteristics, the basic problems of epilepsy control and side effects cannot be ignored.

Our study shows that the therapeutic effect of PB among adult women in rural areas is considerable. The percentage of patients with a seizure-free status in the first year of follow-up was 45.3%, and it steadily increased with longer follow-up times. In the tenth year, 96.6% of patients (*n* = 59) were free of seizures. In a study of PB treatment with a follow-up period of 4 years in Southern India, the proportion of terminal remission ranged from 58% to 66% in patients with good compliance and fewer than 30 seizures. The corresponding range for patients who were not compliant and had more than 30 seizures was 6% to 16% (22). In a study conducted in Mali, 80.2% of patients experienced no seizures for at least 5 months after a 1-year treatment with PB (23). In China, a third of the patients who completed a 1-year treatment were seizure free (15). In another study, 39% of the participants had been seizure free for at least the previous year (24). In our study, the proportion of adult women with seizure-free status was similar or slightly higher than in those previous reports. This rate increased significantly in the tenth year of follow-up, which may be related to the following reasons: patients with good therapeutical effect are more inclined to adhere to PB treatment and follow-up, while those with relatively poor effect may tend to terminate the medication early on their own and withdraw from this project or switch to other drug treatment. The very low number of patients followed for 10 years is also a bias factor. All of the above may contribute to our overestimation of the seizure-free rate. But overall speaking, independent of the side effects, PB is still very suitable as a therapeutic option for women from rural areas with a relative scarcity of medical resources.

We analyzed the risk factors associated with the occurrence of seizures in the first, the third, and the fifth year through multivariate logistic regression. For these different time nodes, the number of seizures in the 12 months before initiation of PB treatment is an important risk factor for seizure occurrence, which has already been suggested by many previous studies. A high initial seizure frequency (more than one per month) before treatment was identified as a predictive factor for seizure recurrence in a study carried on in China (25) and it was also highly associated with intractable epilepsy in newly diagnosed children (26). Long-term and repeated seizures represent the severity of epilepsy to some extent. Patients with recurrent seizures may present progressive sclerosis of the hippocampus and abnormal sprouting of mossy fibers in the dentate gyrus, resulting in ectopic synaptic connections and recurrent excitatory loops that cause recurrent seizures (27). Therefore, seizure frequency at baseline could be a potential indicator of short- and long-term prognosis, which may in turn influence clinical decisions (28).

Higher daily doses at the end of the first year of treatment are an important predictor for seizure recurrence in the first year and the fifth year. In the early stages of treatment, a younger age at onset (1st year outcome) and a high frequency of loss of consciousness during seizures (1st and 3rd year outcome) are also risk factors. All of these may be related to severe conditions. Although some authors believe that generalized tonic-clonic seizures are more likely than partial seizures to be relieved (25), seizures that include loss of consciousness may be more severe. Consciousness depends on the participation of a wide range of brain regions. Many studies have found that in epileptic seizures with impaired consciousness, the changes in brain activity are spread over multiple brain regions (29, 30). These may lead to changes of synaptic connections and excitatory loops, and affect the prognosis of epilepsy.

Many studies have shown that drowsiness is a common side effect of PB treatment (31, 32), the daytime excessive sleepiness could be more frequent in patients with epilepsy (32–34), drowsiness may be associated with PB and seizures, but maybe more often the side effect of PB. However, the relationship between drowsiness and prognosis is not clear. In this study, a higher daily dose of PB is associated with non-seizure-free in the first year, recurrent seizures may lead to higher doses and more severe side effects. Therefore, drowsiness may be also associated with an increased risk of non-seizure-free in the first year.

There have been reports linking PB treatment with a high proportion of side effects related to cognitive performance (35–37), sedation (38), bone health (39), teratogenicity (40), and so on, which may be dose-dependent, resulting in early therapy termination in western countries. However, a series of studies in developing countries have shown that PB has a high degree of tolerability. Other side effects of PB have been observed, but most of them are mild, with drowsiness and dizziness being the most common (31, 41). Among women, the proportion of adverse reactions is slightly higher than that among the entire population (31) and elderly population (41). The proportion of withdrawal due to adverse reactions was as high as 15.4%. Although PB therapy is still tolerated for the treatment of epilepsy in areas where medical resources are scarce, the adverse effects of PB in this population cannot be ignored. We should improve the assessment of the patient's condition as much as possible and administer an appropriately lower dose to control the seizures and reduce at the same time the occurrence of adverse reactions.

In our study, poor compliance was still the main reason for withdrawal, followed by relocation and subsequent loss of contact with the patient. Compared with previous studies that focused on the entire population (15, 42), the proportion of women that withdrew due to side effects and pregnancy planning was higher. The regression analysis showed that younger patients were more likely to withdraw. Younger women may be more sensitive to potential side effects than older people, in particular, because they can be assumed to be reproductively active. This highlights the importance of strengthening education about epilepsy in this setting. Alternative drugs with a lower risk of side effects could also be offered to young people to boost treatment adherence.

Death in our study cohort was closely associated with older age at the time of enrolment. Cardiovascular and cerebrovascular diseases were the most common causes of death, which is consistent with what is observed in the general population of Northeast China. Older women with epilepsy have more age-related underlying diseases. Previous studies have shown epilepsy to be linked to cardiovascular and cerebrovascular diseases (43) and dementia (44), although the causal relationship remains to be explored. Nevertheless, early treatment can still be beneficial for patients and ameliorate the irreversible damage caused by chronic seizures. In past studies, sudden unexpected death in epilepsy (SUDEP) was considered to be an important cause of death (45, 46), and it is the most serious consequence of epilepsy. However, this cause of death was not found in our study, probably because we only collected adult female patients with convulsive epilepsy, rather than all epilepsy patients of all ages and genders. Also, the lack of witnesses to some patients' death and the fact that autopsies are not available for all patients may have contributed to a low detection rate.

There are also some limitations in the present study that cannot be neglected. The quality of the data obtained from door-to-door interviews was affected by the level of expertise of public health workers in the rural areas where the study was conducted. The concentration of the drug, liver function and other parameters related to adverse reactions could not be confirmed by laboratory tests, and neither could the cause of death when it occurred. Due to the lack of medical resources and EEG examination, the specific type of some seizures cannot be clearly identified, so further discussion on this aspect is lacking. Finally, variables related to pregnancy and childbirth were not included in the information obtained from patients due to content limitations on the initial design for the project. Large-scale research on this important aspect is essential for determining the safety of PB treatment in pregnant women who do not have access to alternative treatments and should be carried out in the future.

## Conclusions

PB has high effectiveness, retention rate, and tolerability in adult women with convulsive epilepsy from rural areas. Although its side effects are not negligible, most of them are mild. The baseline seizure frequency before treatment is an important predictor of prognosis. Regular treatment should be provided as early as possible to improve its effectiveness and avoid the aggravation of symptoms. Although the full impact of the drug on pregnancy and related issues warrants further study, PB treatment still has an important role in the management of epilepsy in women living in poverty-stricken areas.

## Data Availability Statement

The raw data supporting the conclusions of this article will be made available by the authors, without undue reservation.

## Ethics Statement

The studies involving human participants were reviewed and approved by the Ethics Committee of the First Hospital of Jilin University. The patients/participants provided their written informed consent to participate in this study.

## Author Contributions

WL guided the design of the study and revised the manuscript. CC designed the study, performed statistical analysis, and wrote the manuscript. NL and RZ gave supporting of analysis and methodology. DZ collected the data. All authors contributed to the article and approved the submitted version.

## Funding

This work was financially supported by the Global Campaign Against Epilepsy demonstration project in rural China (No. 2012027).

## Conflict of Interest

The authors declare that the research was conducted in the absence of any commercial or financial relationships that could be construed as a potential conflict of interest.

## Publisher's Note

All claims expressed in this article are solely those of the authors and do not necessarily represent those of their affiliated organizations, or those of the publisher, the editors and the reviewers. Any product that may be evaluated in this article, or claim that may be made by its manufacturer, is not guaranteed or endorsed by the publisher.

## References

[1] Moshe SL Perucca E Ryvlin P Tomson T. Epilepsy: new advances. Lancet. (2015) 385:884–98. 10.1016/S0140-6736(14)60456-6 25260236

[2] Fiest KM Sauro KM Wiebe S Patten SB Kwon CS Dykeman J. Prevalence and incidence of epilepsy: a systematic review and meta-analysis of international studies. Neurology. (2017) 88:296–303. 10.1212/WNL.0000000000003509 27986877 PMC5272794

[3] Savic I. Sex differences in human epilepsy. Exp Neurol. (2014) 259:38–43. 10.1016/j.expneurol.2014.04.009 24747359

[4] Banerjee PN Filippi D Allen Hauser W. The descriptive epidemiology of epilepsy-a review. Epilepsy Res. (2009) 85:31–45. 10.1016/j.eplepsyres.2009.03.003 19369037 PMC2696575

[5] Herzog AG Fowler KM Sperling MR Massaro JM Progesterone Trial Study G. Distribution of seizures across the menstrual cycle in women with epilepsy. Epilepsia. (2015) 56:e58–62. 10.1111/epi.12969 25823700

[6] Reddy DS. The neuroendocrine basis of sex differences in epilepsy. Pharmacol Biochem Behav. (2017) 152:97–104. 10.1016/j.pbb.2016.07.002 27424276

[7] Kerr C Nixon A Angalakuditi M. The impact of epilepsy on children and adult patients' lives: development of a conceptual model from qualitative literature. Seizure. (2011) 20:764–74. 10.1016/j.seizure.2011.07.007 21831672

[8] Social aspects of epilepsy in the adult in seven European countries. The RESt-1 Group. Epilepsia. (2000) 41:998–1004. 10.1111/j.1528-1157.2000.tb00285.x 10961627

[9] Beghi E. Addressing the burden of epilepsy: many unmet needs. Pharmacol Res. (2016) 107:79–84. 10.1016/j.phrs.2016.03.003 26952026

[10] Ngugi AK Kariuki SM Bottomley C Kleinschmidt I Sander JW Newton CR. Incidence of epilepsy: a systematic review and meta-analysis. Neurology. (2011) 77:1005–12. 10.1212/WNL.0b013e31822cfc90 21893672 PMC3171955

[11] Meinardi H Scott RA Reis R Sander JW World ICotD. The treatment gap in epilepsy: the current situation and ways forward. Epilepsia. (2001) 42:136–49. 10.1046/j.1528-1157.2001.32800.x 11207798

[12] Meyer AC Dua T Ma J Saxena S Birbeck G. Global disparities in the epilepsy treatment gap: a systematic review. Bull World Health Organ. (2010) 88:260–6. 10.2471/BLT.09.064147 20431789 PMC2855595

[13] Wang WZ Wu JZ Wang DS Dai XY Yang B Wang TP. The prevalence and treatment gap in epilepsy in China: an ILAE/IBE/WHO study. Neurology. (2003) 60:1544–5. 10.1212/01.wnl.0000059867.35547.de 12743252

[14] Hu J Si Y Zhou D Mu J Li J Liu L. Prevalence and treatment gap of active convulsive epilepsy: a large community-based survey in rural West China. Seizure. (2014) 23:333–7. 10.1016/j.seizure.2014.01.007 24507246

[15] Wang WZ Wu JZ Ma GY Dai XY Yang B Wang TP. Efficacy assessment of phenobarbital in epilepsy: a large community-based intervention trial in rural China. The Lancet Neurol. (2006) 5:46–52. 10.1016/s1474-4422(05)70254-4 16361022

[16] Aspray TJ. The use of verbal autopsy in attributing cause of death from epilepsy. Epilepsia. (2005) 46(Suppl. 11):15–7. 10.1111/j.1528-1167.2005.00402.x 16393173

[17] Yasiry Z Shorvon SD. How phenobarbital revolutionized epilepsy therapy: the story of phenobarbital therapy in epilepsy in the last 100 years. Epilepsia. (2012) 53(Suppl. 8):26–39. 10.1111/epi.12026 23205960

[18] Brodie MJ Kwan P. Current position of phenobarbital in epilepsy and its future. Epilepsia. (2012) 53(Suppl. 8):40–6. 10.1111/epi.12027 23205961

[19] Christian CA Reddy DS Maguire J Forcelli PA. Sex differences in the epilepsies and associated comorbidities: implications for use and development of pharmacotherapies. Pharmacol Rev. (2020) 72:767–800. 10.1124/pr.119.017392 32817274 PMC7495340

[20] Christensen J Kjeldsen MJ Andersen H Friis ML Sidenius P. Gender differences in epilepsy. Epilepsia. (2005) 46:956–60. 10.1111/j.1528-1167.2005.51204.x 15946339

[21] Hoffman A Levy G. Gender differences in the pharmacodynamics of barbiturates in rats. Pharm Res. (1989) 6:976–81. 10.1023/a:1015953715346 2574444

[22] Mani KS Rangan G Srinivas HV Sridharan VS Subbakrishna DK. Epilepsy control with phenobarbital or phenytoin in rural south India: the Yelandur study. The Lancet. (2001) 357:1316–20. 10.1016/s0140-6736(00)04516-5 11343735

[23] Nimaga K Desplats D Doumbo O Farnarier G. Treatment with phenobarbital and monitoring of epileptic patients in rural Mali. Bull World Health Organ. (2002) 80:532–7. 12163916 PMC2567553

[24] Kwan P Wang W Wu J Li S Yang H Ding D. Long-term outcome of phenobarbital treatment for epilepsy in rural China: a prospective cohort study. Epilepsia. (2013) 54:537–42. 10.1111/epi.12022 23163288

[25] Su L Di Q Kwan P Yu N Zhang Y Hu Y. Prediction for relapse and prognosis of newly diagnosed epilepsy. Acta Neurol Scand. (2013) 127:141–7. 10.1111/j.1600-0404.2012.01711.x 22881868

[26] Berg AT Shinnar S Levy SR Testa FM Smith-Rapaport S Beckerman B. Early development of intractable epilepsy in children: a prospective study. Neurology. (2001) 56:1445–52. 10.1212/wnl.56.11.1445 11402099

[27] Huberfeld G Blauwblomme T Miles R. Hippocampus and epilepsy: findings from human tissues. Rev Neurol. (2015) 171:236–51. 10.1016/j.neurol.2015.01.563 25724711 PMC4409112

[28] Zhu LN Chen D Deng Y He J He YJ Zhou D. Long-term seizure, comorbidity and socioeconomic outcomes of patients with convulsive epilepsy in rural West China. Epilepsy Res. (2020) 168:106480. 10.1016/j.eplepsyres.2020.106480 33120304

[29] Dabrowska N Joshi S Williamson J Lewczuk E Lu Y Oberoi S. Parallel pathways of seizure generalization. Brain. (2019) 142:2336–51. 10.1093/brain/awz170 31237945 PMC6658865

[30] Guo JN Kim R Chen Y Negishi M Jhun S Weiss S. Impaired consciousness in patients with absence seizures investigated by functional MRI, EEG, and behavioural measures: a cross-sectional study. Lancet Neurol. (2016) 15:1336–45. 10.1016/s1474-4422(16)30295-2 27839650 PMC5504428

[31] Si Y Liu L Tian L Mu J Chen D Chen T. A preliminary observation of the adverse effects of phenobarbital among patients with convulsive epilepsy in rural West China. Epilepsy Behav. (2016) 54:65–70. 10.1016/j.yebeh.2015.11.007 26655451

[32] Pizzatto R Lin K Watanabe N Campiolo G Bicalho MA Guarnieri R. Excessive sleepiness and sleep patterns in patients with epilepsy: a case-control study. Epilepsy Behav. (2013) 29:63–6. 10.1016/j.yebeh.2013.06.029 23939029

[33] Maganti R Hausman N Koehn M Sandok E Glurich I Mukesh BN. Excessive daytime sleepiness and sleep complaints among children with epilepsy. Epilepsy Behav. (2006) 8:272–7. 10.1016/j.yebeh.2005.11.002 16352471

[34] Chen NC Tsai MH Chang CC Lu CH Chang WN Lai SL. Sleep quality and daytime sleepiness in patients with epilepsy. Acta Neurol Taiwan. (2011) 20:249–56. 22315175

[35] Vining EP Mellitis ED Dorsen MM Cataldo MF Quaskey SA Spielberg SP. Psychologic and behavioral effects of antiepileptic drugs in children: a double-blind comparison between phenobarbital and valproic acid. Pediatrics. (1987) 80:165–74. 3112727

[36] Farwell JR Lee YJ Hirtz DG Sulzbacher SI Ellenberg JH Nelson KB. Phenobarbital for febrile seizures–effects on intelligence and on seizure recurrence. N Engl J Med. (1990) 322:364–9. 10.1056/NEJM199002083220604 2242106

[37] Chen YJ Kang WM So WC. Comparison of antiepileptic drugs on cognitive function in newly diagnosed epileptic children: a psychometric and neurophysiological study. Epilepsia. (1996) 37:81–6. 10.1111/j.1528-1157.1996.tb00516.x 8603630

[38] Iivanainen M Savolainen H. Side effects of phenobarbital and phenytoin during long-term treatment of epilepsy. Acta Neurol Scand Suppl. (1983) 97:49–67. 10.1111/j.1600-0404.1983.tb01535.x 6424397

[39] Verrotti A Coppola G Parisi P Mohn A Chiarelli F. Bone and calcium metabolism and antiepileptic drugs. Clin Neurol Neurosurg. (2010) 112:1–10. 10.1016/j.clineuro.2009.10.011 19913352

[40] Tomson T Battino D Perucca E. Teratogenicity of antiepileptic drugs. Curr Opin Neurol. (2019) 32:246–52. 10.1097/WCO.0000000000000659 30664067

[41] Li N Li J Zhao D Lin W. Efficacy of phenobarbital in treating elderly epilepsy patients in rural northeast China: a community-based intervention trial. Seizure. (2021) 89:93–8. 10.1016/j.seizure.2021.05.006 34034063

[42] Li J Yang D Zhao D Li N Lin W. Efficacy of phenobarbital and sodium valproate in treating convulsive epilepsy in rural northeast China. Seizure. (2019) 71:207–13. 10.1016/j.seizure.2019.06.012 31394367

[43] Wall J Knight J Emsley HCA. Late-onset epilepsy predicts stroke: Systematic review and meta-analysis. Epilepsy Behav. (2021) 115:107634. 10.1016/j.yebeh.2020.107634 33334717

[44] Subota A Pham T Jette N Sauro K Lorenzetti D Holroyd-Leduc J. The association between dementia and epilepsy: a systematic review and meta-analysis. Epilepsia. (2017) 58:962–72. 10.1111/epi.13744 28397967

[45] Lhatoo SD Sander JW. Cause-specific mortality in epilepsy. Epilepsia. (2005) 46(Suppl. 11):36–9. 10.1111/j.1528-1167.2005.00406.x 16393177

[46] Mu J Liu L Zhang Q Si Y Hu J Fang J. Causes of death among people with convulsive epilepsy in rural West China: a prospective study. Neurology. (2011) 77:132–7. 10.1212/WNL.0b013e318223c784 21653888

