# Supplementary material for: Efficacy of Phenobarbital and Prognosis Predictors in Women With Epilepsy From Rural Northeast China: A 10-Year Follow-Up Study
Source: Front Neurol. 2022 Feb 16;13:838098. doi: 10.3389/fneur.2022.838098 (PMC8889069; doi:10.3389/fneur.2022.838098)
Supplement: Supplementary file 1 [file Table_1.docx]

Supplementary Table

Proportion of different degrees of adverse events between different prognosis, N (%)

|  | In the first year | |  |  | In the third year | |  |  | In the fifth year | |  |  |
| --- | --- | --- | --- | --- | --- | --- | --- | --- | --- | --- | --- | --- |
|  | **Seizure-free**  **(n=548)** | **Non-Seizure-free (n=662)** | **z** | ***P* value** | **Seizure-free**  **(n=722)** | **Non-Seizure-free (n=246)** | **z** | ***P* value** | **Seizure-free**  **(n=703)** | **Non-Seizure-free(n=142)** | **z** | ***P* value** |
| Adverse events |  |  |  |  |  |  |  |  |  |  |  |  |
| Drowsiness |  |  | -3.242 | **0.001** |  |  | -0.070 | 0.944 |  |  | -2.153 | **0.031** |
| None | 295(53.8%) | 300(45.3%) |  |  | 266(36.8%) | 92(37.4%) |  |  | 204(29.0%) | 56(39.4%) |  |  |
| Mild | 246(44.9%) | 338(51.1%) |  |  | 436(60.4%) | 144(58.5%) |  |  | 475(67.6%) | 80(56.3%) |  |  |
| Moderate | 5(0.9%) | 21(3.2%) |  |  | 14(1.9%) | 9(3.7%) |  |  | 16(2.3%) | 4(2.8%) |  |  |
| Serious | 2(0.4%) | 3(0.5%) |  |  | 6(0.8%) | 1(0.4%) |  |  | 8(1.1%) | 2(1.4%) |  |  |
| Ataxia |  |  | -.570 | 0.569 |  |  | -0.079 | 0.937 |  |  | -2.494 | **0.013** |
| None | 447(81.6%) | 532(80.4%) |  |  | 511(70.8%) | 173(70.3%) |  |  | 446(63.4%) | 106(74.6%) |  |  |
| Mild | 94(17.2%) | 118(17.8%) |  |  | 195(27.0%) | 69(28.0%) |  |  | 242(34.4%) | 33(23.2%) |  |  |
| Moderate | 7(1.3%) | 9(1.4%) |  |  | 12(1.7%) | 3(1.2%) |  |  | 11(1.6%) | 3(2.1%) |  |  |
| Serious | 0(0.0%) | 3(0.5%) |  |  | 4(0.6%) | 1(0.4%) |  |  | 4(0.6%) | 0(0.0%) |  |  |
| Dizziness |  |  | -2.260 | **0.024** |  |  | -2.002 | **0.045** |  |  | -2.427 | **0.015** |
| None | 381(69.5%) | 419(63.3%) |  |  | 398(55.1%) | 118(48.0%) |  |  | 314(44.7%) | 82(57.7%) |  |  |
| Mild | 159(29.0%) | 232(35.0%) |  |  | 308(42.7%) | 120(48.8%) |  |  | 373(53.1%) | 53(37.3%) |  |  |
| Moderate | 8(1.5%) | 10(1.5%) |  |  | 14(1.9%) | 8(3.3%) |  |  | 14(2.0%) | 6(4.2%) |  |  |
| Serious | 0(0.0%) | 1(0.2%) |  |  | 2(0.3%) | 0(0.0%) |  |  | 2(0.3%) | 1(0.7%) |  |  |
| Headache |  |  | -1.780 | 0.075 |  |  | -0.756 | 0.450 |  |  | -1.944 | 0.052 |
| None | 434(79.2%) | 496 (74.9%) |  |  | 474(65.7%) | 155(63.0%) |  |  | 404(57.5%) | 96(67.6%) |  |  |
| Mild | 110(20.1%) | 158(23.9%) |  |  | 235(32.5%) | 86(35.0%) |  |  | 288(41.0%) | 40(28.2%) |  |  |
| Moderate | 4(0.7%) | 6(0.9%) |  |  | 11(1.5%) | 4(1.6%) |  |  | 8(1.1%) | 5(3.5%) |  |  |
| Serious | 0(0.0%) | 2(0.3%) |  |  | 2(0.3%) | 1(0.4%) |  |  | 3(0.4%) | 1(0.7%) |  |  |
| Hyperactivity |  |  | -0.147 | 0.883 |  |  | -0.886 | 0.376 |  |  | -2.249 | **0.025** |
| None | 488(89.1%) | 592(89.4%) |  |  | 589(81.6%) | 207(84.1%) |  |  | 542(77.1%) | 122(85.9%) |  |  |
| Mild | 59(10.8%) | 62(9.4%) |  |  | 126(17.5%) | 36(14.6%) |  |  | 153(21.8%) | 17(12.0%) |  |  |
| Moderate | 0(0.0%) | 6(0.9%) |  |  | 3(0.4%) | 3(1.2%) |  |  | 3(0.4%) | 3(2.1%) |  |  |
| Serious | 1(0.2%) | 2(0.3%) |  |  | 4(0.6%) | 0(0.0%) |  |  | 5(0.7%) | 0(0.0%) |  |  |
| Skin rash |  |  | -0.104 | 0.918 |  |  | -1.089 | 0.276 |  |  | -1.902 | 0.057 |
| None | 514(93.8%) | 622(94.0%) |  |  | 630(87.3%) | 221(89.8%) |  |  | 601(85.5%) | 130(91.5%) |  |  |
| Mild | 34(6.2%) | 38(5.7%) |  |  | 89(12.3%) | 25(10.2%) |  |  | 100(14.2%) | 11(7.7%) |  |  |
| Moderate | 0(0.0%) | 1(0.2%) |  |  | 1(0.1%) | 0(0.0%) |  |  | 0(0.0%) | 1(0.7%) |  |  |
| Serious | 0 (0.0%) | 1(0.2%) |  |  | 2(0.3%) | 0(0.0%) |  |  | 2(0.3%) | 0(0.0%) |  |  |
| Gastrointestinal complaints |  |  | -0.277 | 0.782 |  |  | -0.369 | 0.712 |  |  | -1.079 | 0.281 |
| None | 501(91.4%) | 602(90.9%) |  |  | 607(84.1%) | 209(85.0%) |  |  | 562(79.9%) | 119(83.8%) |  |  |
| Mild | 42(7.7%) | 56(8.5%) |  |  | 107(14.8%) | 36(14.6%) |  |  | 132(18.8%) | 22(15.5%) |  |  |
| Moderate | 1 (0.2%) | 2(0.3%) |  |  | 2(0.3%) | 1(0.4%) |  |  | 2(0.3%) | 1(0.7%) |  |  |
| Serious | 4(0.7%) | 2(0.3%) |  |  | 6(0.8%) | 0(0.0%) |  |  | 7(1.0%) | 0(0.0%) |  |  |
| Anxiety or depression |  |  | -1.999 | 0.046 |  |  | -1.318 | 0.187 |  |  | -1.516 | 0.130 |
| None | 498(90.9%) | 577(87.2%) |  |  | 598(82.8%) | 194(78.9%) |  |  | 557(79.2%) | 104(73.2%) |  |  |
| Mild | 46(8.4%) | 84()12.7% |  |  | 117(16.2%) | 52(21.1%) |  |  | 140(19.9%) | 38(26.8%) |  |  |
| Moderate | 3(0.5%) | 0(0.0%) |  |  | 3(0.4%) | 0(0.0%) |  |  | 2(0.3%) | 0(0.0%) |  |  |
| Serious | 1(0.2%) | 1(0.2%) |  |  | 4(0.6%) | 0(0.0%) |  |  | 4(0.6%) | 0(0.0%) |  |  |

Supplement to Table 3
